# Supplementary material for: Interferon-γ-Producing CD4+ T Cells Drive Monocyte Activation in the Bone Marrow During Experimental Leishmania donovani Infection
Source: Front Immunol. 2021 Sep 7;12:700501. doi: 10.3389/fimmu.2021.700501 (PMC8453021; doi:10.3389/fimmu.2021.700501)
Supplement: Supplementary file 1 [file DataSheet_1.pdf]

Supplementary Figure 1

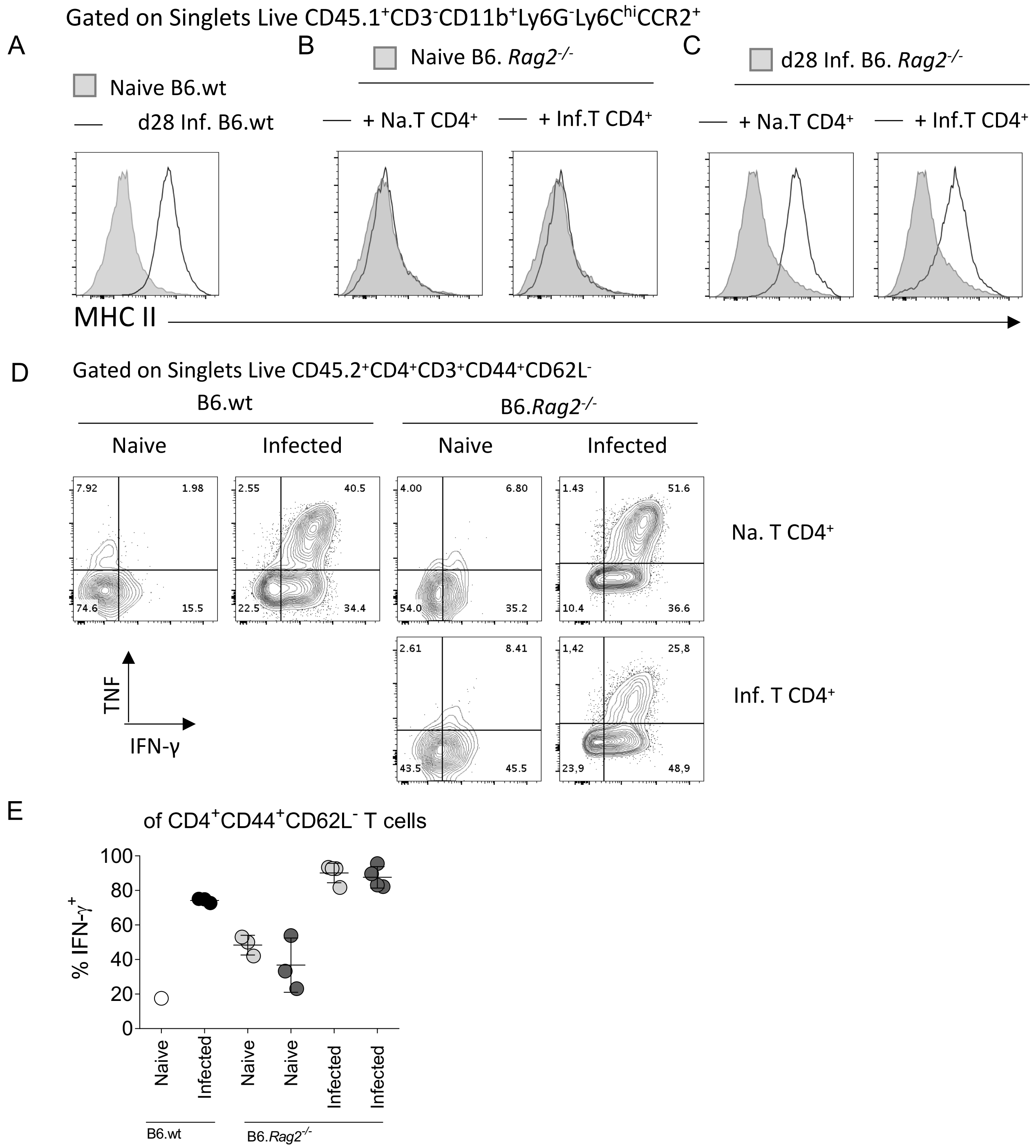

Figure S1 : CD4 T cells fail to activate iMO in absence of *L. donovani* infection. BM CD4+ T cells from naive (Na.T CD4+) or infected (Inf.T CD4+) B6.wt mice were adoptively transfered to naive B6.*Rag2*$^{-/-}$ **A-C.** MHCII expression on iMo in the BM (**A**) of naive and d28 infected B6.wt mice, of naive (**B**) and d28 infected (**C**) B6.*Rag2*$^{-/-}$ 2 weeks post adoptive transfer shown as representative histogram plots. **D.** Cytokine production by adoptively transfered BM CD4+ T cells 2 weeks post transfer into recipient mice shown as representative dot plots. Cells were stimulated with PMA/ionomycin. **E**. Percentage of IFNγ produced by BM CD4+ T cells. Data are derived from analysis of 3 to 5 individual mice of each strain per group and are shown as mean ± SD. Data are representative of 2 independant experiments.

Supplementary Figure 2

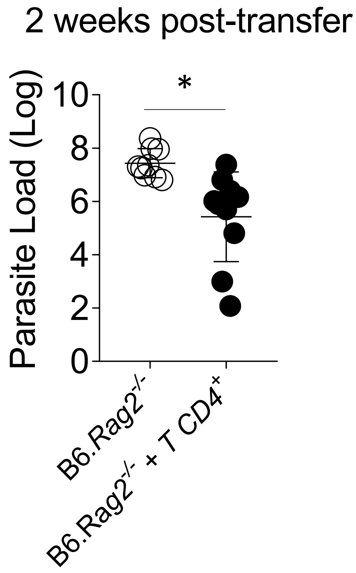

Figure S2 : Adoptive transfer of BM CD4 T cells reduces parasite load in B6.*Rag2$^{-/-}$* mice Parasite load per one million cells in the bone marrow measured by limiting dilution assay. Data are derived from analysis of 8 and 10 individual mice in B6.*Rag2$^{-/-}$* group and in B6.*Rag2$^{-/-}$*+ T $CD4^{+}$ cells respectively. Data are pooled from 3 independent experiments at 2 weeks post transfer are shown as mean ± SD. *, $p<0.05$

Supplementary Figure 3

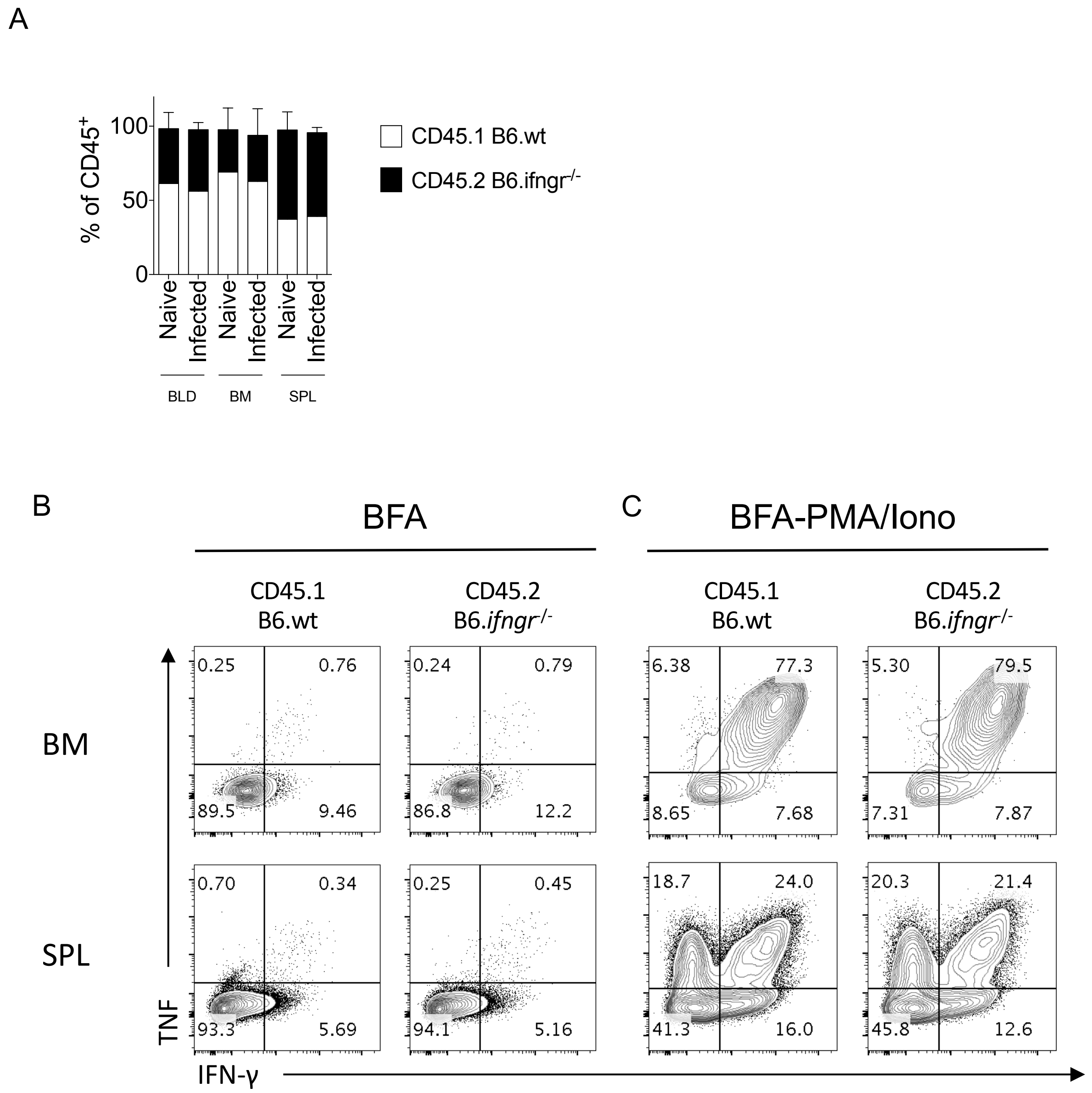

Figure S3 : Cytokine production from CD4$^+$ T cells in mixed bone marrow chimeras

Naïve lethally irradiated B6.CD45.1 recipient mice received a 50:50 of BM cells from B6.CD45.1 and B6.*ifngr1*$^{-/-}$ CD45.2 mice. Mice were subsequently infected with 3x10^7 *L. donovani* amastigotes for 28 days. **A**. Percentage of CD45.1 and CD45.2 of singlets live CD45$^+$ cell population in the blood, BM and spleen. **B-C**. Cytokine production by CD4$^+$ T cells in the BM and the spleen. Cells were stimulated with (**B**) BFA alone or (**C**) PMA/Ionomycin. Data are the pool of 5 mice/group/experiment. Mean +/- SD is shown.

Supplementary Table S1

Statistic Fig 2G		
Comparison	P-value	
B6.wt vs. B6.Rag2-/-	<0.0001	****
B6.wt vs. B6.Rag2-/- + TCD8+	<0.0001	****
B6.wt vs. B6.Rag2-/-+ TCD4+	0.0048	**
B6.wt vs. B6.Rag2-/-Na = <0.0001	<0.0001	****
B6.Rag2-/- vs. B6.Rag2-/- + TCD8+	0.1066	ns
B6.Rag2-/- vs. B6.Rag2-/-+ TCD4+	<0.0001	****
B6.Rag2-/- vs. B6.Rag2-/-Na	0.0033	**
B6.Rag2-/- + TCD8+ vs. B6.Rag2-/-+ TCD4+	<0.0001	****
B6.Rag2-/- + TCD8+ vs. B6.Rag2-/-Na	<0.0001	****
B6.Rag2-/-+ TCD4+ vs. B6.Rag2-/-Na	<0.0001	****

Statistic Fig 4H		
Comparison	P-value	
B6.wt (d14) vs. B6.il10-/- (d14)	0.2572	ns
B6.wt (d14) vs. B6.wt (d56)	0.0095	**
B6.wt (d14) vs. B6.il10-/- (d56)	0.9839	ns
B6.il10-/- (d14) vs. B6.wt (d56)	0.0135	*
B6.il10-/- (d14) vs. B6.il10-/- (d56)	0.1853	ns
B6.wt (d56) vs. B6.il10-/- (d56)	0.0003	****

Statistic Fig 4J

Comparison	P-value	
**B6.wt (d14) vs. B6.il10-/- (d14)**	0.3509	ns
B6.wt (d14) vs. B6.wt (d56)	0.1506	ns
B6.wt (d14) vs. B6.il10-/- (d56)	0,3567	ns
B6.il10-/- (d14) vs. B6.wt (d56)	0,8977	ns
B6.il10-/- (d14) vs. B6.il10-/- (d56)	0,0013	**
B6.wt (d56) vs. B6.il10-/- (d56)	0,0032	**
